# Serum prolidase activity is associated with non-diabetic metabolic syndrome

**DOI:** 10.1186/1758-5996-6-142

**Published:** 2014-12-17

**Authors:** Suzan Tabur, Elif Oguz, Mehmet Ali Eren, Hakan Korkmaz, Esen Savas, Nurten Aksoy, Tevfik Sabuncu

**Affiliations:** Faculty of Medicine, Department of Internal Medicine, Division of Endocrinology, Gaziantep University, 27100, Sahinbey, Gaziantep, Turkey; Faculty of Medicine, Department of Medical Pharmacology, Harran University, 63300 Sanliurfa, Turkey; Faculty of Medicine, Department of Internal Medicine, Division of Endocrinology, Harran University, 63300 Sanliurfa, Turkey; Faculty of Medicine, Department of Internal Medicine, Gaziantep University, 27100 Sahinbey, Gaziantep Turkey; Faculty of Medicine, Department of Clinical Biochemistry, Harran University, 63300 Sanliurfa, Turkey

**Keywords:** Metabolic syndrome, Non-diabetic, Obesity, Prolidase

## Abstract

**Objective:**

The aim of this study was to determine the role of serum prolidase activity and the possible association with oxidative stress parameters in non-diabetic metabolic syndrome.

**Methods:**

30 obese patients without metabolic syndrome (MetS), 34 non-diabetic obese patients with MetS, and 23 volunteer control subjects were enrolled in the study. Fasting plasma glucose (FPG), plasma glucose following 75 g glucose administration, high-density lipoprotein- cholesterol (HDL-C), high-density lipoprotein- cholesterol (LDL-C), total cholesterol, triglyceride (TG), total antioxidant status (TAS), total oxidative status (TOS), oxidative stress index (OSI), and prolidase activities of all subjects were analyzed.

**Results:**

Prolidase levels was significantly higher in MetS group compared to both obese and control groups (p < 0.001 and p < 0.05 respectively). Prolidase was also higher in the obese group than in the control group (p < 0.05). Prolidase was negatively correlated with TAS and HDL-C (r = −0,362, p < 0.001; r = −0.320, p < 0.01, respectively) and positively correlated with BMI, weight, waist-c, SBP, DBP, TG, TC, LDL-C.

**Conclusion:**

Prolidase activity may have a role in the pathogenesis of metabolic syndrome.

## Introduction

Metabolic syndrome (MetS) is defined as the existence of obesity, insulin resistance, glucose intolerance, hypertension, and dyslipidemia [1]. Subjects with MetS may be obese but all obese patients may not have MetS. Both MetS and obesity have been shown to have impacts on cardiovascular mortality and morbidity [2].

Endothelial disfunction causes alterations in the arterial vasculature and leads to micro- and macrovascular complications. The remodelling of the endothelial basal membrane, resulted with erosion and thrombosis, increases the oxidative stress and alters matrix metalloproteinases (MMPs) expression [3].

Prolidase, a member of the MMP family, is a cytosolic imidodipeptidase, which specifically splits imidodipeptides with C-terminal proline or hydroxyproline. The enzyme plays an important role in the recycling of proline from imidodipeptides for resynthesis of collagen and other proline containing proteins [4]. Prolidase enzyme activity has been shown in plasma, erythrocytes, leukocytes, dermal fibroblasts and various organs such as kidney, brain, heart, thymus, uterus, lung, spleen and pancreas [5, 6]. It is demonstrated that the activity of this enzyme may have a role in various disorders such as chronic liver disease, osteoporosis, osteoarthritis, uraemia, and hypertension [7–11]. To the best of our knowledge, there is no data concerning the serum prolidase activity in metabolic syndrome. Therefore, the aim of this study was to determine the role of serum prolidase activity in non-diabetic metabolic syndrome.

## Method

### Subjects

Patients who were admitted for the evaluation of obesity were recruited from the Endocrinology and Internal Medicine outpatient clinic. A standard 75 g oral glucose tolerance test (OGTT) was administered to all participants, and patients were randomized to three groups according to their affected glucose metabolism. Groups included 30 obese patients without MetS and glucose intolerance (mean age 33.67 ± 7.9 years, 2M and 28F), 34 non-diabetic obese patients with MetS (mean age 35.18 ± 6.8 years, 3M and 31F), and 23 sex and age- matched healthy control subjects (mean age32.39 ± 4.7 years, 3M and 20F).

Although the MetS group was composed of non-diabetics, all the patients had varying degrees of glucose intolerance or were insulin resistant. The control group had normal OGTT. MetS is defined according to the criteria accepted in the Third Report of the National Cholesterol Education Program (NCEP) [12]. Hypertension and hyperlipidemia were diagnosed for the first time at the initiation of the study, so no participant was using an anti-hypertensive or anti-lipidemic drug before obtaining the blood samples. Subjects having diabetes, heart failure, cirrhosis, infection, renal failure, pregnancy or malignancy; those on antioxidants such as antihypertensive medications, lipid-lowering medications, and vitamin E; and smokers were excluded.

Age, weight, height, body mass index (BMI: body weight (kg)/height (cm)^2^), and systolic (SBP) and diastolic blood pressures (DBP) of all subjects were recorded. Fasting plasma glucose (FPG), plasma glucose following 75 g glucose administration, high density lipoprotein- cholesterol (HDL-C), Low density lipoprotein-cholesterol (LDL-C), total cholesterol (TC), triglyceride (TG), total antioxidant status (TAS), total oxidative status (TOS), oxidative stress index (OSI), and prolidase activities of all subjects were analyzed. The study was approved by the local ethics committee, and all participants gave signed informed consent.

### Blood samples and preparation

Blood samples were drawn after overnight fasting, and serum samples were stored at −80 °C until biochemical determination of TAS, TOS and prolidase activities.

### Measurement of total antioxidant status

Serum TAS was determined using a novel automated measurement method developed by Erel [12]. In the method, hydroxyl radical, the most potent biological radical, is produced first. In the assay, reagent 1 containing ferrous ion solution is mixed with reagent 2, which contains hydrogen peroxide. The sequentially produced radicals, such as brown colored dianisidinyl radical cation produced by the hydroxyl radical, are also potent radicals. The anti-oxidative effect of the study sample against the potent-free radical reactions, which are initiated by the produced hydroxyl radical, is measured. The assay has excellent precision values, lower than 3%, and the results are expressed as mmol Trolox Equiv./l.

### Measurement of total oxidant status

Serum TOS was determined using a novel automated measurement method developed by Erel [13]. Oxidants present in the study sample oxidize the ferrous ion-o-dianisidine complex to ferric ion. The oxidation is enhanced by glycerol molecules, which are abundantly present in the reaction medium. The ferric ion makes a colored complex with xylenol orange in an acidic medium. The color intensity, which can be measured spectrophotometrically, is related to the total amount of oxidant molecules present in the sample. The assay is calibrated with hydrogen peroxide, and the results are expressed as μmol H_2_O_2_ Equiv./l.

### Oxidative stress index

Percent ratio of TOS to TAS level was accepted as OSI (OSI (Arbitrary Unit) = TOS (μmol H_2_O_2_ Equiv./l)/TAS (mmol Trolox Equiv./l)) [10].

### Prolidase measurement

Prolidase activity was determined by a photometric method based on the measurement of the proline levels produced by prolidase [14].

Serum samples (100 μl) were mixed with 100 μl of serum physiological. A total of 25 μl of the mixture was preincubated with 75 ml of the preincubation solution (50 mmol/l Tris HCl buffer pH 7.0 containing 1 mmol/l glutathione, 50 mmol/l MnCl_2_) at 37°C for 30 min. The reaction mixture, which contained 144 mmol/l gly-pro, pH 7.8 (100 ml), was incubated with 100 ml of preincubated sample at 37°C for 5 min. To stop the incubation reaction, 1 ml glacial acetic acid was added. After adding 300 ml Tris HCl buffer, pH 7.8, and 1 ml ninhydrin solution (3 g/dl ninhydrin was melted in 0.5 mol/l orthophosphoric acid), the mixture was incubated at 90°C for 20 min and cooled with ice. Absorbance was then measured at a 515 nm wavelength to determine proline value.

Intraassay and interassay coefficient of variations (CVs) were lower than 10%. We measured the serum prolidase activity by the method optimized by Gültepe [15], which is a modification of Myara and Chinard's methods [16, 17] based on the spectrophotometric determination of proline levels liberated from glycyl-l-proline by prolidase enzyme.

Plasma TG, total cholesterol, LDL-C, and HDL-C concentrations were measured by automated chemistry analyzer (Aeroset, Abbott) using commercial kits (Abbott).

### Statistical analysis

Continuous variables were expressed as mean ± S.D, and non-parametric data were expressed as median and ranges. Categorical data were compared by Chi-square tests. One-way ANOVA was used for multiple comparisons among the groups, and the LSD test was used if any statistical significance was found. Normality of distribution was evaluated with the Kolmogorov–Smirnov test. Pearson correlation test was used to evaluate any relationships between parameters. All statistical tests were two-sided. P <0.05 was regarded as significant for all analysis. All analyses were conducted using SPSS 11.5 (SPSS for Windows 11.5, Chicago, IL, USA).

## Results

Mean ages of the three groups were similar. SBP, DBP and TG levels were significantly higher in the MetS group compared to both obese and control groups (all p < 0.001) (Table 1). Both obese and MetS groups had significantly higher BMI levels than the control group (all p < 0.001 and p < 0.001 respectively). The obese group had higher SBP and DBP than the control group (all p < 0.001). MetS group had significantly lower HDL-C levels than the control group (p < 0.001). HDL-C levels was also lower in the obese group than in the control group, but the difference was not significant. In the MetS group 10 patients had only impaired fasting glucose (IFG), 2 impaired glucose tolerance (IGT), 9 had both IFG and IGT and 13 were only insülin resistant. Prolidase levels were significantly higher in MetS group compared to both obese and control groups (p < 0.001 and p < 0.05respectively) and also in the obese group compared to the control group (p < 0.05). TAS was lower in both MetS and obese groups than in the control group (p < 0.001 and p < 0.05 respectively). There wasn’t any significant difference according to BMI levels between MetS and obese groups. OSI was significantly higher in both obese and MetS groups than in the control group (p < 0.001 and p < 0.001 respectively). The clinical and biochemical data are shown in Table 1.

**Table 1:** **Clinical and metabolic parameters of MetS, Obese and control groups**

	MetS (n = 34, 31M and 3F)	Obese (n = 30, 28M and 2F)	Control (n = 23, 20M and 3F)
Age (years)	35.18 ± 6.8	33.67 ± 7.9	32.39 ± 4.7
Body weight (kg)	97.27 ± 12.6^b,f^	92.07 ± 19.2^c^	60.30 ± 10.3
BMI (kg/m2)	38.91 ± 5.5^b^	36.59 ± 5.2^c^	22.93 ± 3.4
Waist-C (cm)	106.85 ± 10.3^b,f^	99.73 ± 13.2^c^	75.52 ± 8.1
SBP (mmHg)	136.77 ± 12.0^a,b^	118.67 ± 13.1^c^	105.65 ± 7.7
DBP (mmHg)*	90(70–110)^a,b^	80(70–100)^c^	60(50–80)
TC (mg/dl)	194.39 ± 35.6^e^	181.66 ± 24.2	168.13 ± 24.7
HDL (mg/dl)	44.39 ± 13.3b	42.14 ± 9.9	55.65 ± 5.2
TG (mg/dl)	174.91 ± 72.9^a,b^	100.07 ± 35.3	73.44 ± 30.6
LDL (mg/dl)	113.68 ± 37.4	117.76 ± 23.4^h^	97.80 ± 21.9
TOS (mmol H2O2 Equiv./l)	13.94 ± 2.19^e,f^	12.74 ± 2.1	13.05 ± 2.3
TAS (mmol Trolox Equiv./l)	0.95 ± 0.1^a,b^	1.10 ± 0.1^h^	1.16 ± 0.1
OSI (arbitrary unit)	14.67 ± 2.2^a,b^	11.48 ± 2.3	10.65 ± 2.4
FPG (mg/dl)	102.88 ± 13.3^b^	97.94 ± 7.4^h^	90.87 ± 9.8
Prolidase	708.93 ± 10.4^b,f^	703.17 ± 8.1^h^	696.69 ± 11.5

BMI, body mass index; DBP, diastolic blood pressure; FPG, fasting plasma glucose; HDL, high-density lipoprotein; LDL, low-density lipoprotein; MetS, metabolic syndrome; OSI, oxidative stress index SBP, systolic blood pressure; TAS, total antioxidant status; TC, total cholesterol;TG, triglyceride; TOS, total oxidative status; Waist-C, waist circumference. *Data in which non-parametric tests were used and expressed as median (range). P < 0.001: ^a^MetS versus obese; ^b^MetS versus control; ^c^obese versus control. P < 0.01: ^d^MetS versus obese; ^e^MetS versus control. P < 0.05: ^f^MetS versus obese; ^g^MetS versus control; ^h^obese versus control.

In correlation analysis, prolidase was negatively correlated with TAS and HDL-C (r = −0,362, p < 0.001; r = −0.320, p < 0.01) and positively correlated with BMI, weight, waist-c, SBP, DBP, TG, TC, LDL-C ( r = 0.330, p = 0.002; r = 0.298 p = 0.006; r = 0.4 p <0.001; r = 0.367, p = 0.001; r = 0.358, p = 0.001; r = 0.293, p = 0.008; r = 0.297, p = 0.006; r = 0.306, p = 0.005, respectively; Table 2). These associations were confirmed in the multiple regression analysis ( R^2^ = 0,226, p = 0,001). In multivariate logistic regression analysis prolidase activity was found to be an important predictor for MetS (A one unit change in prolidase would make the MetS 1,115 as likely to ocur; R^2^ = 0,324, p = 0,001).

**Table 2:** **Correlations between prolidase, and other clinical and metabolic parameters**

	BMI	WEIGHT	WAIST-C	SBP	DBP	TG	TC	HDL-C	LDL-C	TAS
PROLIDASE										
*R*	0.330	0.298	0.400	0.367	0.358	0.293	0.297	−0,320	0,306	−0,362
*P*	0.002	0.006	<0.001	,001	,001	,008	,006	,003	,005	,001
BMI										
*R*		0.925	0.892	0.591	0.675	0.423	0.245	−0.304	0.170	−0.437
*P*		<0.001	<0.001	<0.001	<0.001	<0.001	0.023	0.004	0.125	<0.001
WEIGHT										
*R*			0.915	0.581	0.669	0.427	0.311	−0.302	0.234	−0.406
*P*			<0.001	<0.001	<0.001	<0.001	0.004	0.005	0.034	<0.001
WAIST-C										
*R*				0.594	0.675	0.481	0.322	−0.292	0.220	−0.434
*P*				<0.001	<0.001	<0.001	0.002	0.006	0.046	<0.001
SBP										
*R*					0.844	0.436	0.298	−0.197	0.161	−0.494
*P*					<0.001	<0.001	0.005	0.069	0.147	<0.001
DBP										
*R*						0.382	0.333	−0.217	0.218	−0.409
*P*						<0.001	0.002	0.044	0.048	<0.001
TG										
*R*							0.351	−0.166	−0.033	−0.379
*P*							0.001	0.133	0.767	<0.001
TK										
*R*								−0.006	0.849	−0.326
*P*								0.954	<0.001	0.002
HDL-C										
*R*									−0.338	0.123
*P*									0.002	0.264
LDL-C										
*R*										−0.225
*P*										0.042

BMI, body mass index; DBP, diastolic blood pressure; HDL, high-density lipoprotein; LDL, low-density lipoprotein; MetS, metabolic syndrome; SBP, systolic blood pressure; TG, triglyceride; Waist-C, waist circumference; TAS, total antioxidant status.

## Discussion

In this study, we investigated the possible association between serum prolidase activity and non-diabetic metabolic syndrome.We found a significant increase in serum prolidase activity, a member of MMPs, in patients with non-diabetic metabolic syndrome compared to obese or healthy control groups. A significant correlation of serum prolidase activity was also determined both with increased BMI, weight, waist-c, SBP, DPB, TG, TC, LDL-C and decreased TAS and HDL-C levels.

MetS is defined as the existance of obesity and at least other two factors among hypertension, dyslipidemia and diabetes mellitus or glycemia of >100 mg/dl [3]. Endothelial dysfunction in MetS leads cardiovascular risk accompained by high morbidity and mortality. Increased oxidative stress and altered MMPs are shown two of the factors that play in the pathogenesis of MetS. Prolidase, a member of MMPs, plays an important role in collagen metabolism and extracellular matrix remodeling [4, 18]. Prolidase enzyme activity has been investigated in various disorders such as chronic liver disease [7], osteoporosis [8], osteoarthritis [9], uremia [10], diabetic neuropathy [19], hypertension [11], coronary artery disease [20], and ovarian cancer [21]. There are some studies revealing the role of MMPs in MetS. Goncalves et al. reported an increase in pro-MMP-9, MMP-8 and TIMP-1 levels while without any difference in MMP-2, MMP-3 and TIMP-2 levels compared to healthy controls [22]. Additionally, an increase in MMP-8 levels in MetS patients [23] and elevated levels of MMP-2 activity, but not of MMP-9 in non-diabetic MetS [24] was reported. On the other hand, there are some studies in the literature regarding MMPs profile in obesity [24–29], diabetes mellitus [30–33] hypertension [34–36] and dyslipidemia [37, 38], clinical conditions representing diagnostic criteria for the definition of the metabolic syndrome.

We have shown previously that MetS and obesity may alter oxidative stress, which contributes to atherosclerosis-related cardiovascular events [39]. In this study we also found a significant increase (p < 0.001) of OSİ levels and a significant decrease (p < 0.001) of TAS levels in metabolic syndrome and non-diabetic Mets group compared to obese and healthy control groups similar to our previous study [39].

We eveluated firstly serum prolidase activity in non-diabetic metabolic syndrome and demonstrated its elevation in this patient group. Additionally, the correlation analysis showed that prolidase activity had a significant positive correlation with BMI, weight, waist-c, SBP, DBP, TG, TC, LDL-C and inversely negative correlation with TAS and HDL-C in our study. Correlation between serum prolidase activity and markers of oxidative stress paremeters in this study suggests the association of collagen turnover and oxidative stress in non-diabetic MetS.

Serum prolidase activity was significantly higher in MetS group compared to the only obese group. This may be resulted from that hypertension, hypertriglyceridemia, low HDL-C levels, IGT, IFG and insülin resistance, are found more frequently in MetS compared to obesity. Logistic regression analysis demonstrated that prolidase activity was an important predictor for MetS as for the last point of this study.

Demirbağ et al. [11] has found a significant correlation between prolidase activity and presence and duration of hypertension supporting our data. Yıldız et al. [20] also showed that serum prolidase activity was positively correlated with presence of hypertension, SBP and inversely correlated with HDL-C levels. Hilali et al. [18] reported that elevated serum prolidase activity and oxidative stress may be associated with increased cardiovascular risk in polycystic ovary syndrome and/or menstrual irregularities associated with this syndrome. Serum prolidase activity was suggested as a marker of osteoporosis in type 2 diabetes mellitus [8].

Consequently we suggest that evaluating prolidase activity in subjects with non-diabetic MetS may be important as an independent predictor of the disease. However, further studies in larger patient groups are needed to explain the role of serum prolidase activity in the pathogenesis of metabolic syndrome.

## Abbreviations

MetS: Metabolic syndrome
MMPs: Matrix metalloproteinases
OGTT: Oral glucose tolerance test
NCEP: National Cholesterol Education Program
BMI: Body mass index
SBP: Systolic blood pressures
DBP: Diastolic blood pressures
FPG: Fasting plasma glucose
TG: Triglyceride
TAS: Total antioxidant status
TOS: Total oxidative status
OSI: Oxidative stress index
HDL-C: High density lipoprotein- cholesterol
LDL-C: Low density lipoprotein-cholesterol
IFG: Impaired fasting glucose
IGT: Impaired glucose tolerance
TIMP: Tissue inhibitors of metalloproteinase.
